# Tribute to Mark Keller

**Published:** 1995

**Figure:**
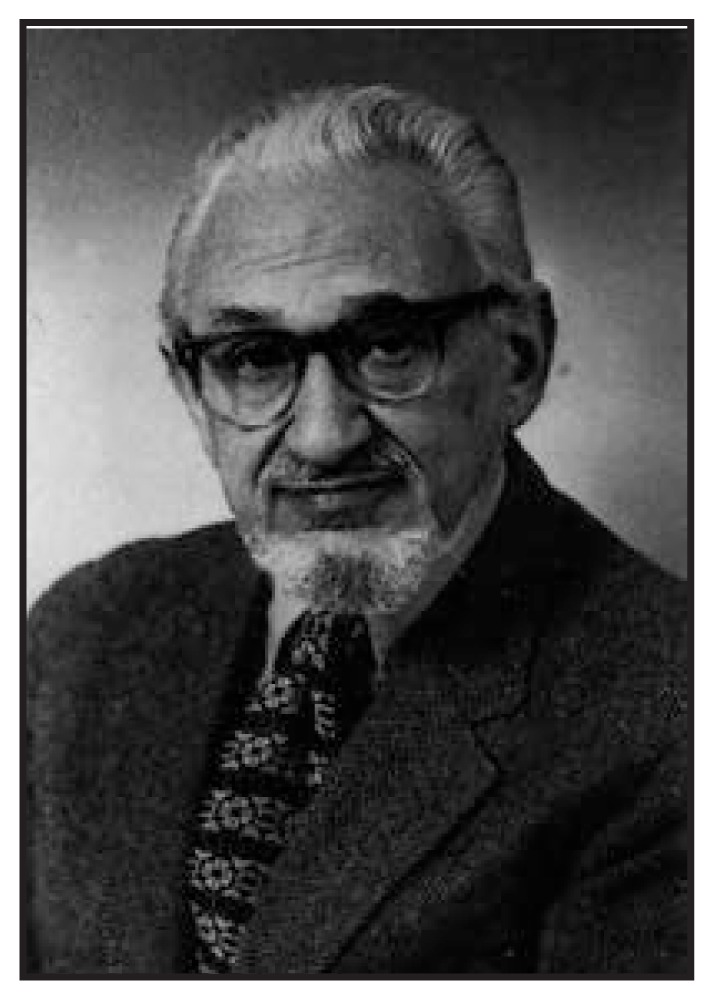


The National Institute on Alcohol Abuse and Alcoholism and the entire alcoholism community were deeply saddened by the death on August 12, 1995, of Mark Keller, professor emeritus of alcohol studies at Rutgers University and a pioneer in the field of alcohol research.

Though principally self-taught, Mr. Keller’s motivation and inquisitiveness resulted in laudable academic achievements. He began his career in alcohol-related research and teaching in the mid-1930’s at the New York University School of Medicine. In the early 1940’s, at Yale University, he helped found the first Center of Alcohol Studies. He and the Center then moved to Rutgers University in 1962. There, Mr. Keller helped develop the Center’s unique library, which continues to be one of the most complete alcohol-relevant research and reference libraries in the world. He also shared his extensive knowledge of the field through lectures at Brandeis University.

Mr. Keller’s more than 200 publications are testament to his lifelong commitment to bringing order to the field of alcohol research, as evidenced by his text, the *Dictionary of Words about Alcohol*. He also edited the *International Bibliography of Studies on Alcohol* and was editor of the first and second volumes of the *Special Report to the U.S. Congress on Alcohol and Health*. For the better part of 50 years, as editor and, later, as editor emeritus, he supported and guided the publication of the *Journal of Studies on Alcohol*. After his official retirement, Mr. Keller continued to pursue his work as a scholar and lecturer, and he recently was studying interpretations of the historical aspects of the Biblical texts.

In 1991 Mr. Keller coauthored an article for *Alcohol Health & Research World* on defining alcoholism. In an interview in that issue, Mr. Keller was asked how he viewed the future of alcohol research. He responded, “I’m an optimist. My long-range outlook about the field is that gradually, as the result of research, we will learn more and more, and we’ll begin to learn how to prevent alcoholism.”

Thanks to Mr. Keller’s dedication, scholarship, and the unique contributions he made to the field of alcohology (Keller’s term), we are that much closer to realizing this goal.

